# Complementary and Alternative Medicine Use and Its Association with Quality of Life among Cancer Patients Receiving Chemotherapy in Ethiopia: A Cross-Sectional Study

**DOI:** 10.1155/2016/2809875

**Published:** 2016-06-28

**Authors:** Daniel Asfaw Erku

**Affiliations:** Department of Pharmaceutical Chemistry, School of Pharmacy, University of Gondar, Lideta Kebele 16, P.O. Box 196, Gondar, Ethiopia

## Abstract

*Background. *Today, complementary and alternative medicine (CAM) use is being routinely practiced by cancer patients worldwide. This study aimed at examining the prevalence of CAM use in patients with cancer and comparing the quality of life (QoL) in CAM users and nonusers.* Methods.* A cross-sectional study was employed on 195 cancer patients receiving chemotherapy at Gondar University Referral Hospital (GURH) chemotherapy center. Interviewer-administered questionnaires were used and the collected data were analyzed by the Statistical Package for the Social Sciences (SPSS) software version 21.0 for Windows.* Results. *154 (79%) patients were found to be users of CAM. Educational status, average monthly income, disease stage, and comorbidity were strong predictors of use of CAM. The most commonly utilized types of CAM were traditional herbal based medicine (72.1%) and only 20.8% of patients discuss with their doctors CAM use. No significant difference was found in QoL between CAM users and nonusers except in financial difficulties (*p* = 0.020).* Conclusions. *This study revealed a high rate of CAM use with very low disclosure rate to their health care providers. Health care providers should be open to discuss the use of CAM with their patients as it will lead to better health outcome.

## 1. Introduction

Ethiopia is among the most populous African countries with prediction of being the top 10 most populous countries in the world by the year 2050. Currently, cancer is becoming the primary public health issue in the country owing to its fast growing rate [1]. According to International Agency for Research on Cancer 2015 report, annual incidence and mortality of all cancers in Ethiopia were more than 6,500 and 50,000, respectively [2]. Yet, there are only two cancer specialized referral hospitals (Black Lion Hospital and Gondar University Hospital) and there is no organized cancer registry center in the country. Both referral hospitals have a very limited number of oncology specialists and materials with less than 30 beds and a single radiology center. Owing to the poor health care system of Ethiopia, most patients are required to go through many referrals, starting from primary health care centers to referral hospitals. This, coupled with the longer waiting times for treatment, contributed to the presentation of patients with advanced cancer stage. In addition, most patients often first visit traditional healers and seek alternative medicine services rather than conventional medicine [1].

Complementary and alternative medicine (CAM) is defined as a variety of ways including different medical and health care systems, various practices, and many products that are not treated as part of modern conventional medicine [3]. There is a huge body of literature documenting the use of CAM in cancer patients. A recent large population based cross-sectional survey employed regarding CAM use in more than 10 European countries found out that more than two-thirds of adult cancer patients used some form of CAM for alleviating their disease and treatment effects [4]. A review of different studies conducted in western countries also underlined that the overall prevalence of CAM use in cancer patients was around 40% [5]. The prevalence of CAM use in cancer patients in Asia and Malaysia was found to 98% and 60%, respectively [6, 7].

Cancer patients utilizing some form of CAM often seek to improve health and get better quality of life (QoL) [8]. According to World Health Organization (WHO), QoL is defined as “a perception of life, perceived values, and interests in the scaffold of culture.” In western countries, QoL evaluation has become more and more important as health care providers seek to understand the role health care interventions play in patients' lives rather than their physical outcomes [9]. In recent years, studies have been conducted on CAM use and QoL and most of the studies, despite variation in study methods and definition of CAM, reported no statistically significant differences in QoL between CAM consumers and nonconsumers [10–12]. However, some studies reported that CAM users have a lower overall QoL than non-CAM consumers [13, 14].

Despite the huge body of literature published elsewhere in the world regarding CAM use by cancer patients, there is no research article published regarding the prevalence of CAM use and its association with QoL cancer patients in Ethiopia. Taking the global evidence into consideration and due to lack of data in Ethiopia, this study was conducted to assess the prevalence of CAM use in cancer patients and to compare the QoL in CAM consumers and nonconsumers in patients attending Gondar University Referral Hospital (GURH) chemotherapy center.

## 2. Materials and Methods

### 2.1. Study Design and Setting

An institutional based cross-sectional study was employed on cancer patients receiving chemotherapy at GURH chemotherapy center from October 2015 to February 2016. GURH is located in Gondar town, northwest Ethiopia, 738 km away from Addis Ababa (the capital city of Ethiopia). The health care system in Ethiopia is ordered into a 4-tier system, divided into primary health care units, district hospitals, general hospitals, and specialized referral hospitals. GURH is among the oldest and pioneering teaching referral hospitals with a range of specialists including pediatrics, surgery, gynecology, psychiatry, and a recently established oncology center. It is among the two referral hospitals in the country which are specialized in cancer treatment and it is the only cancer treatment center found in Amhara region. The cancer treatment center, having 10 beds, 1 oncologist, 3 surgeons, and 5 nurses dedicated for oncology ward, provides chemotherapy and surgery services for cancer patients living in Gondar town and its surrounding areas. The hospital also refers patients who need radiology treatment to Black Lion Hospital where radiology treatment solely exists in the country.

### 2.2. Population and Sampling

A convenience sample of adult cancer patients who attended GURH chemotherapy center between October 2015 and February 2016 (a total of 231 patients) were invited to participate. Adult (>18 years old) cancer patients regardless of stage and time since diagnosis, who had undergone a minimum of one cycle of standard-dose chemotherapy and who were able to understand the questionnaire and give their consent, were eligible to be included. The exclusion criteria are patients who lack understanding of oral Amharic language, patients who had severe physical or psychological problems, or those who refused to participate.

### 2.3. Data Collection and Management

Data collection was performed by three well-trained nurses through interviewer-administered questionnaires. All cancer patients who attended GURH chemotherapy center between October 2015 and February 2016 and met the inclusion criteria were invited to participate. The questionnaire, originally written in English, was translated to local language (Amharic) and back to English in order to ensure that the translated version gives the proper meaning. The content validity of the tool (questionnaire) was confirmed by a team of experts including a senior physician, epidemiologist, and clinical pharmacist. The questionnaire was pretested on 15 cancer patients prior to the real data collection that were excluded from final study, and relevant modifications were instituted.

### 2.4. Questionnaire

The final questionnaire includes three main parts. Part one included questions that ask information regarding the sociodemographic and treatment characteristics including age, sex, marital status, educational level, cancer site (all cancer types), clinical stage, type of treatment (chemotherapy, surgery, or both), duration since diagnosis, and employment status. The second section of the questionnaire included queries assessing the prevalence of CAM use, information source about CAM, and discussion with physicians about CAM use. The use of CAM among patients was assessed by a series of questions including the following: “do you have a history of CAM use?” And if the answer is yes, respondents were asked, “which of the following CAMs have you used (at least 4 times)?” Participants were labeled as CAM consumers if they had utilized at least one type of CAM for more than 4 times. Four times is suggested as a minimum indicator for dedication in CAM use [15]. Patients were given five categories to choose from and told that more than one choice is possible. The categories were as follows: traditional medicine (herbal based), special foods (honey, black seed, soy, pomegranate, ginger, or others), dietary supplements, spiritual healing (prayers, lighting candles, consuming holy water such as “Tsebel” (a type of holy water for orthodox Christians), and fasting (abstinence from any food or drink)), and miscellaneous (vitamins and minerals supplements or “others”). Types of CAM included were based on prevalent CAM practices reported in Ethiopia identified through literature review [16]. The final part, data regarding QoL, was collected using the Amharic version of EORTC QLQ-C30 version 3 [17]. The EORTC QLQ-C30 questionnaire, originally written in English, has been internationally validated [17–20] and is currently translated into more than 80 languages including Amharic language. Even though the cross-cultural adaptation of the Amharic version is not well established in diverse Ethiopian population, it has been previously used in a study done in Addis Ababa, Ethiopia [21]. The questionnaire includes a global health status, functional scales, and symptom scales. The extent to which the participants experienced symptoms was measured as follows: 1: not at all, 2: a little, 3: quite a bit, and 4: very much. A high score represented a healthy level of functioning and a high QoL, but a high mean score for a symptom scale characterizes a high level of problems.

### 2.5. Statistical Analysis

The final data collection tool was ensured for completeness, and responses were entered into and analyzed by the Statistical Package for the Social Sciences (SPSS) software version 21.0 for Windows. Frequencies and percentages, means with standard deviations were used to describe different variables. The EORTC QLQ-C30 items were scored and linearly transformed to a 0–100 scale according to the EORTC Scoring Manual [22]. The characteristics of CAM consumers and nonconsumers were compared by using Pearson's chi-square test. Associations with significance levels of less than 0.20 (*p* < 0.20) in the univariate analysis were included in the multivariate logistic regression analysis. The results were adjusted for patients' demographic and clinical characteristics. Odds ratio with 95% confidence interval (95% CI) was also computed along with corresponding *p* value (*p* < 0.05).

### 2.6. Ethical Considerations

This study was approved by the ethical committee of University of Gondar. Permission letters were received from EORTC research group to use the instrument. Informed consent from the patients was also obtained before conducting this study. Participants' information obtained was kept confidential.

## 3. Results

### 3.1. Sociodemographic and Clinical Characteristics

Out of the 231 cancer patients invited to participate, 195 completed the questionnaire (response rate: 84.4%). Among 195 patients surveyed, 152 (78%) of respondents need interviewer assistance due to physical inability and inability to read and write. As a result, the data from these patients were collected by three well-trained nurses. The remaining 43 (22%) patients fill in the questionnaires by themselves. The sociodemographic and clinical characteristics of study participants are summarized in Table 1. More than half of the patients 106 (54.3%) were females and the rest 89 (45.6%) were males with a female to male ratio of 1 : 1.9. Out of the 195 patients surveyed, 154 (79%) are CAM users, while 41(21%) were nonusers. There were statistically significant (*p* < 0.001) differences in educational status, average monthly income (*p* value < 0.001), the disease stage (*p* value: 0.013), and presence of comorbidity between CAM consumers and non-CAM consumers (*p* value: 0.020). After controlling for many other variables, educational status, average monthly income, disease stage, and presence of comorbidity remained to be significant in the multivariate logistic model. The odds of use of CAM among patients with average monthly incomes above USD 125 were 5.12 times higher than among patients with average monthly incomes lower than USD 125. The odds for CAM use in patients with higher (tertiary) education were 2.73 times higher than in patients with primary or lower educational level. The odds for CAM use among patients who have comorbid illness were 3.71 times higher than in patients without comorbidity. The odds for CAM use among patients with late-stage cancer were 2.85 times higher than in patients with early-stage cancer.

### 3.2. Type and Characteristics of CAM Use

**Figure 1:**
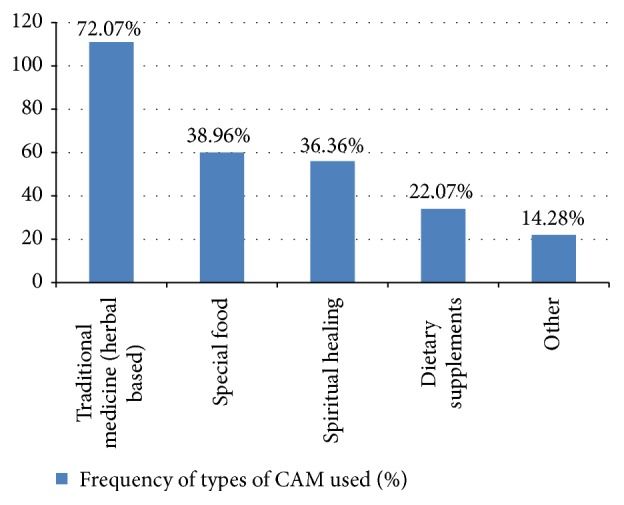
Frequency of different types of CAM used by the study participants (*N* = 195).

The various types of CAM used by patients are illustrated in Figure 1. The most commonly consumed type of CAM was traditional herbal based medicine followed by special foods and spiritual healing. Dietary supplements and others (vitamins and minerals supplements) were rated as fourth and fifth in reported use.

Table 2 describes the characteristics of CAM use among study participants. The most commonly cited source of information about CAM was families, relatives, and friends (46.1%) followed by other cancer patients using CAM (38.3%). The most commonly cited reason for using CAM was “belief in advantages of CAM (23.4%),” followed by “dissatisfaction with conventional therapy (14.9%),” “family tradition/culture (13%),” and “emotional support (11%).” The most cited reasons for not using CAM among nonusers were “lack of belief in the benefits of CAM (39%)” followed by “afraid of side effect (31.7%).” Large proportions (79.2%) of CAM users did not discuss their use of CAM with their physicians. The main motive for not communicating with their doctors was that they thought the doctors have negative response for CAM use (56.5%). Some of the respondents also think that it was not important for doctor to know about their CAM use (22.1%). Most of CAM users (81.8%) did not experience side effects from CAM use and 74% of users stated that they planned to continue their CAM use. Only 9.7% CAM users answered that they were not satisfied with their CAM use.

### 3.3. Association between CAM Use and QoL

Table 3 shows the mean value for each subscale of the EORTC QLQ-C30 questionnaire. After adjusting for different variables, there were no noteworthy variations between CAM users and non-CAM users in global health status and all subscales of the EORTC QLQ-C30 except for financial difficulties, where CAM users (54.86 ± 4.67) had significantly (*p* = 0.020) higher marginal mean for financial difficulties than those who did not use CAM.

## 4. Discussion

Our study revealed that CAM use is common among Ethiopian cancer patients receiving chemotherapy. The prevalence of CAM use reported in our study (79%) is much higher compared to the survey conducted in more than 10 countries of Europe which reported a prevalence of 44.7% [23]. However, our finding is comparable with studies conducted in Malaysia, Canada, and Korea (62.5%, 71.2%, and 74.8%, resp.) [24–26]. The high prevalence of CAM use in our study could be partially explained by the fact that the culture in Ethiopia encourages the use of CAM especially herbal based traditional medicine and spiritual healing. It is also a well-known fact that more than two-thirds of the Ethiopian population depend on traditional medicine for the treatment of their medical condition [16]. The variations in the prevalence of use of CAM across different regions of the globe can be explained by variations in sociocultural background and perceptions of the importance of CAM, differences in the accessibility of western medicine, and differences in the criteria used to define CAM use in various studies. CAM users in our study had a higher monthly household income, attend higher education, were at an advanced stage of cancer, and were suffering from comorbidities. The findings were consistent with studies done in many parts of the globe [27–30] where CAM consumers had higher education, higher income, advanced cancer stage, and comorbid illnesses; all of them have been identified as factors of CAM use in our study. Educated and economically strong patients may be more likely to explore other therapies and ways to muddle through with the disease state and treatment effects [31].

The most commonly used CAM in our study was traditional herbal based medicines followed by special foods and spiritual healing. The elevated prevalence of use of traditional herbal based medicines in the present study can be partially explained by the fact that Ethiopia is endowed with a rich and diverse flora that constituted a basis for primary health care [16]. Furthermore, the prevalent use of these therapies might be due to the common perception that such therapies and practices are natural and does not cause any deleterious effect though it is not scientifically supported. Spiritual healing was also used by a considerable percentage of patients in this study, specifically “prayer” and “holy water.” A common practice to all religions in Ethiopia including Muslims and Christians is the incorporation of religious convictions in daily practices, with prayer and fasting being an integral piece of the culture. In our study, the common information source about CAM (46.1%) was family members, friends, and relatives. In contrast, medical practitioners (2.6%) were the least information source for CAM use. Our finding partially corroborates the study done in Korea [32] which identifies family members and relatives as the common information source about CAM use. This result is also similar to the study done in German, where the most prominent sources of information for CAM choice were outside the medical community and included families, relatives, and friends (49%) [33]. However, few other studies reported media such as internet, television, radio, newspapers, and magazines as the main information source about CAM [34, 35]. To prevent the abuse of CAM, health care providers should have open discussion with their patients about CAM use and provide appropriate information on the safety and efficacy of CAM therapies. In our study, CAM use was discussed with their physicians by only 20.8% of patients using CAM. This is comparable to the 32.7% reported in an earlier study of Korean cancer patients and 29.6% in Malaysian patients with cancer [32, 34]. However, it is much lower than the 71% reported by breast cancer patients in USA [36]. A systematic review of the characteristics of CAM use among breast cancer patients indicated that more than half of patients do not disclose CAM use to their doctors [37]. The major reasons cited for not discussing CAM use with the doctor in the present study were “anticipating negative response about CAM use” and “it was not important for doctor to know about my CAM use.” This could be because of the fact that the general negative attitude of doctors to CAM products and practices may discourage patients from sharing information about their CAM use. In a study done by Tasaki et al. [38], some of the obstacles of communication about CAM were physician antagonism toward CAM use and patient anticipation of discouraging response from their physician. The lack of communication between the physician and patients using CAM may have a harmful effect on patient health status as a result of toxic effect of CAM or to interactions with the modern treatments. Therefore, physicians should acknowledge the use of CAM by their patients, encouraging active conversation for the proper and rational use of CAM.

The findings of this study showed that there were no considerable differences between CAM consumers and non-CAM consumers in QoL. This finding corroborate a study conducted by Chui et al. [10–12] that reported no considerable differences in QoL between CAM consumers and non-CAM consumers in Malaysia and Turkey, respectively. Similarly, a study done in Korea by Kang et al. [34] and in Germany by Tautz et al. [33] found out that the global QoL of breast cancer patients between CAM consumers and nonconsumers was not different. In contrast to our finding, several previous studies [13, 14] found that CAM users had a lower QoL than non-CAM users.

CAM users in our study, however, appeared to experience more financial difficulties than nonusers. Similar findings were also reported in studies done elsewhere [10, 11]. Ethiopia is among one of the poorest countries in the world. The health care cost associated with the treatment for cancer is not usually affordable compared to patients' income (the gross national income per head based on purchasing power parity in the Ethiopia is below US $1000 [1]) as all the costs for cancer care and treatment are usually covered by the patients themselves. Due to this, most patients first seek CAM practice as it is relatively affordable compared to the conventional medicine. The financial load of cancer treatment including the high cost of chemotherapy and additional price of CAM may be the cause for financial difficulties faced by cancer patients. The financial burden faced by CAM users could also be due to the fact that this study was done in Ethiopian public referral hospital, which is more often visited by patients with financial problems (low and middle income patients).

### 4.1. Study Limitations

The study has several limitations that should be taken into account while interpreting the results. First, because the study is conducted in only one chemotherapy center, the results found regarding CAM use may not be representative of all Ethiopian cancer patients. Second, as the study design is cross-sectional in nature, there may be no causal relationships with CAM use. Thirdly, we used EORTIC QLQ-C30 version 3 for assessing QoL of cancer patients. The instruments' reliability and cultural adaptation and validity are not well established in Ethiopia, which may have affected our finding. Assessing QoL using other multilingual QoL measures such as WHOQoL, which is culturally adapted in Ethiopia, would have improved the finding of this study. Finally, among non-CAM users, there may have been patients who may have used CAM less than the minimum required frequency (four times), and this could have underscored the number of CAM users. A larger-scale and multicentered survey that includes more diverse participants is needed to provide more accurate findings.

## 5. Conclusion

The present study confirms that CAM use is prevalent among Ethiopian cancer patients, traditional herbal based medicine, special foods, and spiritual healing being the most commonly used. Patients depend mainly on family, friends, and relatives as a source of information about CAM and majority of patients did not discuss CAM use with their health care provider. This study also showed that there were no considerable differences in QoL between CAM consumers and non-CAM consumers. Doctors should find a way to discuss the use of CAM with their patients as it will lead to less risk of toxicity due to CAM use. Furthermore, health care administrators should give more emphasis to CAM users and give appropriate financial support since CAM users are likely to face financial difficulties.

## Figures and Tables

**Table 1:** Sociodemographic and clinical characteristics of CAM users and non-CAM users.

Variables	CAM users (%) *N* = 154	Non-CAM users (%) *N* = 41	*p* value	Multivariate logistic regression CAM users versus non-CAM users AOR (95% CI)
*Age*			0.754	
18–29	19 (12.3%)	5 (12.2%)		—
30–39	44 (28.6%)	7 (17.1%)		—
40–49	56 (36.4%)	18 (43.9%)		—
50–59	20 (13%)	5 (12.2%)		—
60+	15 (9.7%)	6 (14.6%)		—

*Sex*			0.300	
Male	72 (46.8%)	17 (41.5%)		—
Female	82 (53.2%)	24 (58.5%)		—

*Educational status*			<0.001^*∗*^	
Primary	28 (18.2%)	50 (32.5%)		1
Secondary	76 (49.3%)	77 (50%)		1.35 (0.90–2.29)
Tertiary	50 (32.5%)	27 (17.5%)		2.73 (1.27–4.78)^*∗*^

*Marital status*				
Single	26 (16.9%)	8 (19.5%)	0.632	—
Ever married	128 (83.1%)	33 (80.5%)		—

*Average monthly income*				
<125 USD	109 (70.8%)	35 (85.4%)	0.001^*∗*^	1
>125 USD	45 (29.2%)	6 (14%)		5.12 (1.82–6.05)^*∗*^

*Employment status*			0.162	
Unemployed	108 (70.1%)	30 (73.2%)		1
Employed	46 (29.9%)	11 (26.8%)		0.89 (0.51–1.51)

*Religion*			0.347	
Orthodox	68 (44.1%)	7 (17.1%)		—
Muslim	39 (25.3%)	8 (19.5%)		—
Protestant	21 (13.6%)	10 (24.4%)		—
Catholic	19 (12.3%)	12 (29.3%)		—
Others^*∗∗*^	7 (4.5%)	4 (9.7%)		—

*Cancer type*			0.584	
Hematologic malignancies	50 (32.5%)	11 (26%)		—
Breast cancer	56 (36.4%)	18 (43.9%)		—
Gastrointestinal malignancies^1^	18 (11.7%)	4 (9.7%)		—
Gynecologic^2^ malignancies	18 (11.7%)	6 (14.6%)		—
Others	12 (7.8%)	2 (4.9%)		—

*Duration of cancer *			0.132	
<1 year	65 (42.2%)	15 (36.6%)		1
1–5 year	55 (35.7%)	16 (39%)		0.70 (0.35–1.38)
>5 year	36 (23.4%)	10 (24.4%)		0.80 (0.37–1.72)

*Stage of disease*			0.013^*∗*^	
Early	94 (61%)	29 (70.7%)		1
Advanced	60 (39%)	12 (29.3%)		2.85 (1.73–2.93)^*∗*^

*Treatment modality*			0.300	
Chemotherapy	89 (57.8%)	32 (78%)		—
Surgery	35 (22.7%)	5 (12.2%)		—
Both	30 (19.5%)	4 (9.7%)		—

*Chemotherapy cycle*			0.584	
<4	106 (68.8%)	29 (70.7%)		—
>4	48 (31.2%)	12 (29.3%)		—

*Comorbidity*			0.020^*∗*^	
No	52 (33.8%)	29 (70.7%)		1
Yes	102 (66.2%)	12 (29.3%)		3.71 (1.97–6.53)^*∗*^

^1^Stomach, pancreas, liver, colon, rectum, and anus. ^2^Ovaries, cervix, and other.  ^*∗*^Significant association (*p* value less than 0.05). ^*∗∗*^Jehovah witness, Adventist.

AOR: adjusted odds ratio; USD: united states dollar.

**Table 2:** Prevalence and characteristics of CAM use in the study population (*N* = 195).

Variables about CAM use	Frequency (%)
*CAM use*	
Yes	154 (79%)
No	41 (21%)

*Source of information about CAM *	
Families, friends, and relatives	71 (46.1%)
Health care professionals	4 (2.6%)
Media (internet, television, radio, and book)	14 (9.1%)
Patients using CAM	59 (38.3%)
Others	6 (3.9%)

*Reasons for CAM use *	
Belief in advantages of CAM	36 (23.4%)
Family tradition/culture	20 (13%)
Emotional support	17 (11%)
Boosting immune system	13 (8.4%)
Prevention of recurrence	8 (5.2%)
Dissatisfaction with conventional therapy	23 (14.9%)
Synergic effect of conventional therapy	8 (5.2%)
Decrease side effect of conventional therapy	9 (5.8%)
Treatment of other medical problems	15 (9.7%)
Others	5 (3.2%)

*Reasons for not using CAM among nonusers *	
Lack of belief in the benefits of CAM	16 (39%)
The doctor did not prescribe CAM	6 (14.6%)
Afraid of side effect	13 (31.7%)
Never heard of CAM	2 (4.9%)
Additional burden	4 (9.7%)

*Consult with doctor about CAM use *	
No	122 (79.2%)
Yes	32 (20.8%)

*Reason for not consulting with doctor *	
Anticipating negative response about CAM use	87 (56.5%)
Insufficient information of CAM	11 (7.1%)
No need to consult with doctor	13 (8.4%)
It was not important for doctor to know about my CAM use	34 (22.1%)
Others	9 (5.8%)

*Side effects from CAM *	
No	126 (81.8%)
Yes	28 (18.2%)

*Satisfaction with CAM *	
Satisfied	76 (49.3%)
Average	63 (40.9%)
Dissatisfied	15 (9.7%)

*Would you use CAM again? *	
No	30 (19.5%)
Yes	114 (74%)
Undecided	10 (6.5%)

**Table 3:** Quality of life (EORTC QLQ-C30) scores among CAM users and non-CAM users (*N* = 195).

EORTC QLQ-C30	Non-CAM users	CAM users	*p* value
	Mean ± SD	Mean ± SD	
*Global health status*	61.54 ± 2.34	59.76 ± 2.16	0.583

*Functional scales*			
Cognitive	79.56 ± 3.43	77.49 ± 3.77	0.158
Physical	80.74 ± 1.21	79.21 ± 1.89	0.367
Emotional	71.72 ± 3.15	69.81 ± 2.99	0.454
Role	62.89 ± 2.21	60.34 ± 2.73	0.510
Social	59.43 ± 3.94	58.35 ± 3.89	0.458

*Symptom scales*			
Fatigue	31.46 ± 4.61	34.75 ± 3.37	0.283
Nausea & vomiting	49.72 ± 1.29	51.12 ± 1.99	0.420
Appetite loss	24.71 ± 3.68	24.16 ± 3.37	0.418
Pain	33.08 ± 4.45	36.14 ± 4.13	0.091
Dyspnoea	13.77 ± 1.61	11.41 ± 2.83	0.914
Insomnia	42.78 ± 4.49	46.79 ± 3.95	0.218
Diarrhoea	28.22 ± 1.64	29.44 ± 1.03	0.361
Constipation	29.74 ± 2.32	28.83 ± 2.52	0.131
Financial difficulties	46.27 ± 3.71	54.86 ± 4.67	0.020^*∗*^

^*∗*^Significant association (*p* value less than 0.05).
